# Genetic parameters of production and reproduction traits of Egyptian buffaloes under subtropical conditions

**DOI:** 10.1007/s11250-022-03251-2

**Published:** 2022-08-24

**Authors:** Ahmed A. Easa, Ayman H. Abd El-Aziz, Adel S. A. El Barbary, Nikolay M. Kostomakhin, Mohammed A. F. Nasr, Tharwat A. Imbabi

**Affiliations:** 1 Department of Animal and Poultry Production, Faculty of Agriculture, Damanhour University, Abadiyyat Damanhur, Egypt; 2 Department of Animal Husbandry and Animal Wealth Development, Faculty of Veterinary Medicine, Damanhour University, Abadiyyat Damanhur, Egypt; 3 Department of Animal Production, Faculty of Agriculture, Alexandria University, Alexandria, Egypt; 4 Department of Dairy and Beef Cattle Breeding, Russian State Agrarian University - Moscow Agriculture Academy named After K.A. Timiryazev, Moscow, Russia; 5 Department of Animal Wealth Development, Faculty of Veterinary Medicine, Zagazig University, Sharkia, Egypt; 6 Department of Animal Production, Faculty of Agriculture, Benha University, Benha, Egypt

**Keywords:** Productive performance, Reproductive performance, Egyptian buffalo, Genetic parameters

## Abstract

This research was aimed to investigate the production and reproduction traits and their genetic parameters of Egyptian buffaloes under subtropical environment. Heritability, phenotypic, and genetic parameters were estimated for productive and reproduction traits of first parity This study revealed the averages TMY, 305-dMY, LL, BW, DP, SP, CI, and AFC were 2260, 2150 kg, 271 days, 38.63 kg, 210.50 days, 195.20 days, 586.86 days, and 36.61 months, respectively. The heritability estimates for productive traits of the first lactation were 0.61, 0.52, 0.47, 0.20, and 0.23 for TMY, 305-dMY, LL, DP, and BW, respectively. Heritability estimates for reproductive traits SP, CI, and AFC were 0.07, 0.09, and 0.35, respectively. Genetic correlations of most of the investigated first parity traits were positive, with an exception of DP. The genetic correlation was negative between SP and BW, and between AFC and LL. Phenotypic correlations of the first parity among all investigated traits were positive except between DP and TMY, 305-dMY, LL, BW, and AFC. High and moderate heritability estimates indicated that the opportunity of genetic upgrading of these parameters could be achieved owing to sire selection. Selection for high milk yield will be associated with genetic improvement in lactation period and birth weight.

## Introduction

Buffalo (*Bubalus bubalis*) is subsequent to dairy cows concerning milk yield (Coroian et al., 2013) but gives the highest milk quality of domestic animals (Senosy and Hussein 2013), due to its white colour, high fat level and flavour (Abd El-Salam and El-Shibiny 2011). Buffaloes capable of converting low-quality feed and forage to milk/meat compared to dairy cows (Ibrahim 2012). Therefore, their rearing and production improve the farmer family’s life (Aggarwal and Upadhyay 2013). A short time ago, the need for buffalo milk has increased. Therefore, improving milk production is the leading rationale for selecting dairy cattle to produce more milk (Nasr 2016).

Not only buffalo well known with their resistance to diseases and parasites, long productive life, adaptation to low-quality food, and poor management, but also with inferior reproduction performance (long calving interval and days open, late reproductive maturity with higher age at first calving) (Aziz et al., 2001; Amin et al., 2021; Nasr 2017a,b). Animal genetic configures its fitness and adaptation to critical environment such as high temperature and health troubles. Adaptation is genetically expressed as hereditary of buffalo traits which maintain animal existence (Niyas et al., 2015). Egyptian buffaloes are bearable and stout at tropical and subtropical condition when compared to their crossing with the Italian one (Nasr 2017b). They distinguished by the greater reproductive performance (Nasr 2017c).

The course of improving the buffalo performance is tremendously muddied; for example, selection for a single parameter may give rise to a destructive influence on milk quality along with reproductive traits (Barros et al., 2014). Contrariwise, the heritability value of milk production was moderate, proposing it will be improved by direct mass selection (Malhado et al., 2013). There were several factors that influenced the reproduction, such as animal body condition score, dam age at insemination and insemination season (Grimard et al., 2006; Zobel et al., 2011), milk yield (Grimard et al., 2006; Ledoux et al., 2015), days in milk at insemination, calving to first service (Ledoux et al., 2015), and farm management (Zobel et al., 2011). High yielding animals were in higher risk of early and late fetal death (Abdalla et al., 2017).

There were several results revealed that milk production and fertility was negatively correlated (phenotypic/genetic) (Dunklee 1991; Dematawewa and Berger 1998; Lyons et al., 1991; Pryce et al., 2004). This fact may amplify the semen expenses, veterinary budgets, days open, services per conception, and CI. Commonly, the majority of the reproduction efficiency had (0.10) low heritable estimates (Kadarmideen et al., 2003; Wall et al., 2003). Even though weak heritability of reproduction traits, their additive genetic distinction was enough to approve competent selection for fertility (Weigel and Rekaya 2000). Consequently, this investigation aimed to evaluate heritability, phenotypic, genetic correlations of some production, and reproduction performance of Egyptian buffalos that are of financial consequence to the dairy business.

## Materials and methods

The current data were collected since 1990 to 2017 from buffalo’s dairy herd kept in Mehallet Moussa, Buffalo Research Institute, Ministry of Agriculture, Kafr El- Sheikh Governorate. Data consists of 907 buffaloes and 66 sires. Animals machine milked twice a day and dried off 60 days before the anticipating calving time. Rectal palpation was applied to check the pregnancy. Buffalo heifers mated firstly at 1.5 years of age or at body weight of 350 kg. The amounts of milk produced per buffalo were recorded on daily basis.

The total mixed ration (50 kg/buffalo/day) was provided to animals that meet with the expected needs for productive and reproductive periods. The main diet assessment comprised of crude protein (12.5%), net energy (Mcal k̸g = 2.4), neutral detergent fibre (31%), and acid detergent fibre (19%). This research was conducted on buffaloes between 2nd and 7th parities. The temperatures at the ranch ranged from 11.5 to 20.7 °C in cold months and from 24.1 to 37.4 °C in the hot months. The collected productive traits were total milk yield (TMY), 305-day milk yield (305-dMY), lactation length (LL), birth weight (BW), and dry period (DP), while the reproductive traits were service period (SP), calving interval (CI), and age at first calving (AFC). Animals possessed LL of fewer than 100 days were removed from this study (Hess et al. 2016).

### Statistical analysis

Variance components and genetic parameters were estimated by REML using MTDFREML program (Boldman et al., 1995) as the following:

$$y=Xb+Zu+Wp+e$$

where *y* is observation vector of records, *b* is fixed effects vector (e.g., year of calving, month of calving and parity), *u* is animal direct effect vector, *p* is permanent environmental effect vector, and *e* is residual effect vector, while *X*, *Z*, and *W* were the incidence relating records to fixed, animal, and permanent environmental effects, respectively.

## Results

### Productive and reproductive traits

The current investigation presented that the total milk production and 305-day milk production of Egyptian buffalo were 2269 and 2150 kg/season, respectively with lactation length of 271 days (Table 1). Regarding the reproductive traits, Egyptian buffalo gave calf birth weight of 38.63 kg with dry period of 210 days, service period of 195.20 days, calving interval 586.86 days, and the age at first calving was 36.61 months (Table 1).

**Table 1:** Productive and reproductive traits of Egyptian buffaloes (means, standard deviations (SD), and coefficient of variations (CV%)) with heritability estimates (*h*^2^) and standard errors (SE) for these traits

Traits	Mean ± SD	CV (%)	*h*^2^ ± SE
Total milk yield (kg)	2260 ± 701	31.11	0.61 ± 0.155
305-day milk yield (kg)	2150 ± 495	23.02	0.52 ± 0.143
Lactation length (day)	271 ± 120	44.28	0.47 ± 0.117
Age at first calving (month)	36.61 ± 6.14	15.84	0.35 ± 0.102
Dry period (day)	210.50 ± 106.05	40.03	0.23 ± 0.087
Service period (day)	195.20 ± 75.10	35.15	0.07 ± 0.065
Birth weight (kg)	38.63 ± 5.98	15.48	0.20 ± 0.084
Calving interval (day)	586.86 ± 149.01	25.61	0.09 ± 0.068

### Heritability estimates

There were strong *h*^2^ values for total milk production (0.61), 305-day milk production (0.52), lactation length (0.47), and age at first calving (0.35) (Table 1), while it was moderate for dry period and calf birth weight 0.23 and 0.20, respectively. The heritability estimates were very weak for service period and calving interval 0.07 and 0.09, respectively (Table 1).

### Genetic and phenotypic correlation

Table 2 presents the genetic and phenotypic correlation for production and reproduction performance of Egyptian buffalo. There was a strong positive highly significant genetic correlation between total milk production and each of the following 305-day milk production, lactation length, calf birth weight, service period, calving interval, and age at first calving, while it was negatively correlated with dry period (− 0.61). Dry period was negative genetically correlated with service period, calving interval, and calf birth weight − 0.44, − 0.38, and − 0.50, respectively. Moreover, calf birth weight had a negative highly significant genotypic correlation with service period (− 0.42), with phenotypically positive highly significant correlation with total milk production, 305-day milk production, and LL 0.15, 0.16, and 0.12, respectively (Table 2).

**Table 2:** Genetic correlations (above diagonal) and phenotypic correlations (below diagonal) for productive and reproductive traits in Egyptian buffaloes

Traits	TMY	305-dMY	LL	BW	DP	SP	CI	AFC
TMY	-	0.98**	0.80**	0.26**	− 0.61**	0.99**	0.82**	0.25**
305-dMY	0.86**	-	0.80**	0.33**	− 0.70**	0.88**	0.82**	0.32**
LL	0.79**	0.57**	-	0.48**	− 0.61**	0.84**	0.71**	− 0.24**
BW	0.15**	0.16**	0.12**	-	− 0.50**	− 0.42**	0.05	0.11**
DP	− 0.07*	− 0.05	− 0.07**	− 0.08*	-	− 0.44**	− 0.38**	− 0.13**
SP	0.06	0.05	0.03	0.004	0.35**	-	0.96**	0.33**
CI	0.10**	0.10**	0.05	0.04	0.33**	0.55**	-	0.61**
AFC	0.04	0.02	-0.05	0.12**	− 0.001	0.006*	0.02	-

*TMY* total milk yield, *305-dMY* 305-day milk yield, *LL* lactation length, *BW* birth weight, *DP* dry period, *SP* service period, *CI* calving interval, *AFC* age at first calving

^*^Significant at *P* < 0.05; **significant at *P* < 0.01

## Discussion

The current milk yield of this study was supported by others on the same species (Nasr 2016, 2017a,b,c; Nasr et al., 2017). They reported the average total milk production of Egyptian buffalo was ranged between 1878–2277 kg/season. There were conflicting outcomes regarding the LL of Egyptian buffaloes. Some researchers reported the average lactation length was 238–252 days (Nasr 2017a,b,c; Nasr et al., 2017), while others reported a range of 220–226 days (Mostafa et al., 2017; Shalaby et al., 2016). Our finding was comparable to Barros et al. (2014) who reported a 270-day lactation length in Brazilian buffaloes.

Regarding the age at first calving, Barros et al. (2016) detected that the Murrah buffalo first calved at 37.6 months that was comparable with the current finding on Egyptian buffalo. But, recently, Helmy and Somida (2021) detected 45.16 months for the age at first calving of Egyptian buffalo, while the dry period and service period were logger than that recently reported by others (Nasr 2017a,b; Nasr et al., 2017), who mentioned that the dry period and service period of Egyptian buffalo ranged between 116–147 and 82–107 days, respectively. These periods were 85 days less than that reported by Mourad et al. (1989) on the same breed which reflected a continuous improvement of reproductive and productive performance of Egyptian breed. In the past, the Egyptian buffalo calf birth weight was 28 kg (Kotby et al., 1989; Sharma et al., 1984) and then recently improved to reach 34–37 kg (Abu El-Naser 2020; El-Bramony et al., 2008) and 40–41 kg (Nasr 2017a,b; Nasr et al., 2017). These results supported our finding.

The variation among the published studies regarding the productive and reproductive performance of buffalo may be attributed to (1) biological variation among different populations, (2) statistical methods and sample size used (Silva del Rio et al., 2007), (3) animal supervision (Ghavi Hossein-Zadeh et al., 2008), (4) age and weight of cows and calf birth weight (Hearnshaw and Morris, 1984), (5) herd management practices (Bicalho et al., 2008; Berry et al., 2007), (6) genetically different, (7) herd size, (8) environmental and managerial condition, (9) feed quality, and (10) calving season and order (Araújo et al., 2012; Bhutkar et al., 2014; Fooda et al., 2010; Macedo et al., 2001).

The *h*^2^ estimate for lactation milk production and lactation length in the present study were higher than that reported by others (Barros et al., 2016; El-Arian et al., 2012; Mostafa et al., 2017; Mourad and Khattab 2009; Vij 1986), who stated that it was ranged between 0.24 to 0.55 and 0.13 to 0.41 of Egyptian buffalo, respectively. The *h*^2^ estimate of the age at first calving in the current study was approximately similar to Van der Westhuizen et al. (2001), who mentioned that there was a moderate *h*^2^ value (0.40) for age at first calving in multi-breed beef cows, while it was higher than that reported by Helmy and Somida (2021). Calving interval revealed a very weak *h*^2^ estimate that was comparable to others (Morammazi et al., 2007; Mourad et al., 1989; Ramos et al., 2006; Shalaby et al., 2016). Consequently, the findings of this study designated that these traits might be gradually respond to the direct selection. This agreed with Aby et al. (2012) that pointed out the genetic aptitudes of farms for reproduction performance might be gradually improved by selection. The obtainability of genomic evidence in animals’ genetic assessment has delivered a prospect to boost competence of selection, particularly for modest heritable parameters.

Hammoud (2013) stated a negative genetic correlation between milk yield and days open. On contrast, there was a strong positive genetic correlation between milk production and service period (0.98) (Zink et al., 2012) and (0.46) (Lopez et al., 2019) which supported our finding (0.99). Several researchers detected a negative genetic correlation between milk production and dry period (EL-Arian et al., 2003; Salem et al., 2006; Shalaby et al., 2016) which supported our findings.

There were conflicting results regarding the genetic correlation between AFC and CI. Some authors showed a negative genetic correlation (Cavani et al., 2015; Mercadante et al., 2000), while others reported a positive genetic correlation (Berry and Evans 2014; Lopez et al., 2019; Oyama et al., 2004). Our findings were in conformity with the preponderance of the researchers that AFC was positively correlated with CI. High and positive genetic relationship of age at first calving and calving interval in the current research indicates the buffalo gave her first calf at younger age would have a shorter calving interval. But these estimates have high standard errors that reflected unreliable decision based on this relationship.

The current results agreed with those reported by Barros et al. (2016) and Singh et al. (1990) who stated a significant genetic correlation between total milk yield and age at first calving (0.24–0.28) and was 0.84–0.95 between lactation length and calving interval (Singh and Basu 1988; Singh et al., 1990). The current results showed a significant positive genetic correlation between calf birth weight and milk production which supported by others (Dutt and Yadav 1988; Gogoi et al., 1987). There was a negative genetic correlation between dry period and calving interval that was − 0.76 (Yadav and Rathi 1991), while it was − 0.50 between dry period and service period. These findings supported our results.

## Conclusion

High and moderate heritable estimates for total milk production, 305-day MY, LL, birth weight, and DP indicated that the possibility of genetic improvement of these traits could be achieved through sire selection, while low heritability estimates for reproduction parameters (service period and calving interval) indicated that the most variation in reproductive traits may be due to non–genetic factors. Selection for high milk yield will be associated with genetic improvement in lactation period and birth weight. Moreover, high genetic correlation among total milk yield, 305-dMY, and LL elucidated these parameters were possibly regulated by similar number of genes so that these parameters might be enhanced concurrently via selective breeding program.

## Data Availability

All data generated or analysed during this study are included in this published paper.
